# Effectiveness of Psychosocial Interventions for Adults With Substance Use Disorder That Have a Co‐Occurring Common Mental Health Disorder: An Umbrella Review

**DOI:** 10.1111/dar.70066

**Published:** 2025-11-05

**Authors:** Emma L. Simpson, Munira Essat, Ruth Wong, Sarah Stacey, Edward Day

**Affiliations:** ^1^ University of Sheffield Sheffield UK; ^2^ NHS Inclusion, St George's Hospital Stafford UK; ^3^ University of Birmingham Birmingham UK

**Keywords:** mental disorders, psychosocial intervention, substance‐related disorders, systematic review

## Abstract

**Issues:**

People with substance use disorders can have co‐occurring mental disorders.

**Approach:**

An umbrella review was conducted to identify evidence of the effectiveness of psychosocial interventions for adults (aged 18+) with substance use disorders and co‐occurring common mental health disorders. Systematic reviews were sought of randomised controlled trials of psychosocial interventions compared to each other, treatment as usual or wait‐list. Five databases were systematically searched in February 2024. Data, including critical appraisal (Joanna Briggs Institute Checklist), were extracted by one reviewer and checked by another. Data were discussed in a narrative review.

**Key Findings:**

Of 5420 unique records, 28 systematic reviews were included. The methodological quality of the reviews was good. Most reviews focused on depression, anxiety or post‐traumatic stress disorder. There was much heterogeneity between reviews, and randomised controlled trials within reviews. Most of the interventions and many of the treatment‐as‐usual comparators resulted in significant improvement in substance use and mental health disorders. Results suggested integrated (co‐ordinated) treatment for co‐occurring diagnosis patients was better than treating one condition alone, and usually better than parallel uncoordinated services. There was limited evidence assessing sequential treatment, but this suggested similar effectiveness to integrated treatment.

**Implications:**

Implications for current practise could not be recommended due to heterogeneity. Improvement shown by all types of psychosocial intervention including active comparators precluded recommending one type of intervention over another.

**Conclusion:**

Further research is needed comparing integrated with parallel or sequential treatment, with follow‐up of 6 months or longer, and sample size large enough to encompass dropout.

**Summary**

- Most of the psychosocial interventions studied resulted in significant improvement in substance use and mental health disorders.
- Evidence suggested integrated (co‐ordinated) treatment for co‐occurring diagnosis patients was usually better than parallel uncoordinated services.
- Limited evidence was found on sequential treatment.

## 1 Introduction

People with alcohol or drug use disorders can have a co‐occurring mental disorder. In England (April 2022 to March 2023), there were 290,635 adults in contact with drug and alcohol services [1]. Public Health England [2] found the majority of substance use disorder (SUD) service users had co‐occurring mental health problems (serious mental illness or common mental health disorders [depression, anxiety, post‐traumatic stress disorder, phobias, eating disorders, obsessive compulsive disorder]), with 70% of drug users and 86% of alcohol users reporting comorbid mental health problems. It has been suggested that mood disorders are up to 4.7 times more prevalent in SUD than in the general population [3]. A study of UK patients found that within drug service users, 37% had a personality disorder, 19% had severe anxiety and 67% had depression. Amongst alcohol service users, 53% had a personality disorder, 32% had severe anxiety and 81% had depression [4, 5]. A study of alcohol use in an English population found that for people with phobias, there was a prevalence of 17% hazardous drinking and 9% harmful drinking (probable dependence) [6]. In the population with probable post‐traumatic stress disorder (PTSD), there were prevalences of 18% hazardous and 8% harmful alcohol use [6]. People with eating disorders have a higher rate of SUD than the general population [7].

With co‐occurring diagnosis of SUD and mental health problems, the conditions can aggravate each other, increasing psychiatric symptoms and impairing health‐related quality of life [8, 9, 10]. Comorbid conditions can be difficult to treat. There can be problems for people with comorbid disorders accessing services, where community mental health teams may offer psychological interventions for mental health disorders but may exclude those with substance use disorder, but specialist addiction services may not provide interventions for comorbid mental health disorder. According to service users, barriers to accessing services may include: lack of information about local services; stigma; lack of facilities for a range of interventions; service user difficulties such as with transport or limited finances [11]. People with co‐occurring diagnoses have higher rates of treatment dropout or non‐compliance [12].

The National Institute for Health and Care Excellence has produced guidance on treating people with alcohol or drug use disorders with a co‐occurring serious mental disorder; however this does not cover those where the mental disorder is less severe [13, 14]. Thus there was a need for this review which addressed treatment for those with substance use disorder and co‐occurring common mental disorders.

## 2 Methods

As part of a larger project of clinical and cost‐effectiveness, this umbrella review was reported in accordance with the Preferred Reporting Items for Systematic Review and Meta‐Analysis (PRISMA) [15]. The protocol was registered in PROSPERO registration number CRD42024515813.

### 2.1 Data Sources and Search Strategy

Searching of five electronic databases, MEDLINE, Embase, PsycINFO, Cochrane Database of Systematic Reviews and Web of Science; contact with experts in the field; and scrutiny of bibliographies of retrieved papers. Systematic database searches were carried out in February 2024. Search terms were grouped into one of four concepts: (i) mental health co‐occurrence, co‐occurring or comorbid diagnosis; (ii) patient groups with mental health conditions identified through consultation with the clinical advisors, National Institute of Health and Care Excellence list of mental disorders [16, 17], Improving Access to Psychological Therapies treated mental health conditions [18]; (iii) general terms for substance misuse and psychoactive substances; and (iv) named substances. The MEDLINE search strategy is provided as an Appendix A.

### 2.2 Study Selection

Inclusion criteria for the population were: Adult patients (age 18 years or over) with a moderate to severe substance (alcohol or drugs) use disorder (SUD), including harmful substance use [19, 20], who have a co‐occurring common mental health disorder (depression, anxiety, post‐traumatic stress disorder, phobias, eating disorders, obsessive compulsive disorder). Included interventions were psychosocial interventions (with or without adjunctive pharmacological therapies) for SUD with co‐occurring mental disorder; compared with other psychosocial treatments, treatment as usual, wait‐list or no treatment. Included settings were health or social care services in countries with similar services to the UK. Reviews had to report substance use and mental health outcomes. Due to the breadth of the inclusion criteria, systematic reviews of randomised controlled trials (RCT) were sought, published in English in peer‐reviewed journals. Reviews were excluded if the population had severe mental illness (psychosis, schizophrenia, bipolar disorder). Borderline personality disorder was excluded (although it formed part of the larger project funded by the National Institute for Health and Care Research [11]). Smoking cessation or detoxification services were excluded. Where there were multiple systematic reviews of the same topic, the most recent review was included if this incorporated all relevant RCTs across those reviews [21]. However, for cases where there were overlapping but non‐identical review questions, and the reviews included some of the same primary studies, both reviews were included and the amount of overlap of primary studies was stated [22, 23, 24]. Where systematic reviews had a broader population than our eligibility criteria, they were eligible for inclusion if either SUD outcome or mental disorder outcome data were summarised separately for the population meeting our review.

A 5% sample of the records retrieved by electronic searches was checked by two reviewers, and in the case of high agreement (as measured by Cohen's kappa *k* = 0.8 or higher) [25], one reviewer assessed the rest of the records. There was the option to check further 5% samples until reviewers reached an agreement. The full texts of selected records were obtained and assessed against the inclusion criteria. Study selection based on full texts involved a 10% sample check by two reviewers with discrepancies resolved by discussion, with the option to check further samples until agreement was reached. Following this, one reviewer conducted the remaining full text study selection.

### 2.3 Quality Assessment

Critical appraisal of included reviews was performed by one reviewer and checked by a second reviewer, using the Joanna Briggs Institute Critical Appraisal Checklist for Systematic Reviews and Research Syntheses.

### 2.4 Data Extraction and Synthesis

A standardised form was constructed, including review characteristics, results and the reviews' risk of bias assessment of included RCTs. Where there were several editions of the same systematic review, the most up‐to‐date edition was used. Where studies had wider populations or comparators outside the scope of this review, only reviews allowing a summary evidence for the relevant subgroup were included, and only these data for the relevant subgroup were extracted [23]. Data were extracted by one reviewer and checked by a second reviewer. Interventions were grouped into categories. Any disagreements were resolved through discussion and consultation with a clinical advisor where necessary. Data were tabulated and discussed in a narrative review, by conducting a preliminary synthesis by organising results into categories, mapping evidence and exploring relationships in the data (based on methods guidance by Popay et al. [26]).

## 3 Results

### 3.1 Review Characteristics

**FIGURE 1:**
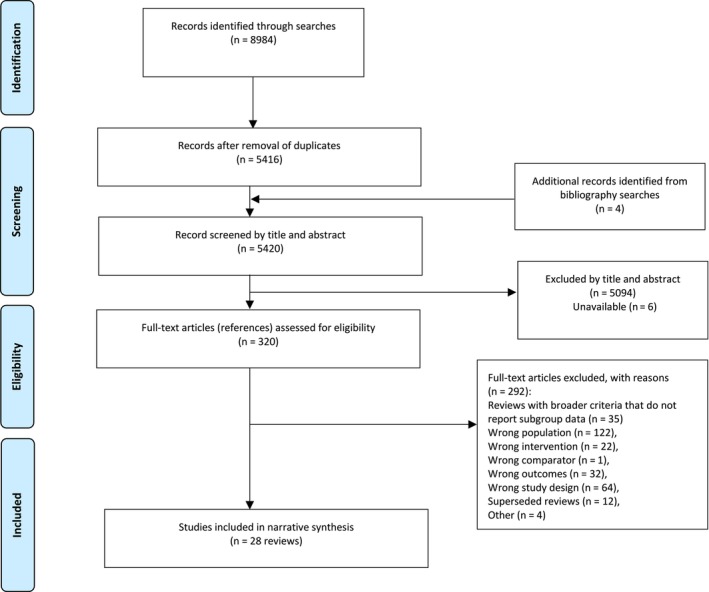
PRISMA flow diagram.

Database searches identified 5416 unique records, and an additional four records were added from bibliography searching. Two reviewers independently title and abstract sifted 280 records. There was an agreement of 93.9%, with Cohen's kappa *k* = 0.9. Twenty‐eight systematic reviews of clinical effectiveness were included (Figure 1). Most reviews focused on depression, anxiety or post‐traumatic stress disorder (Tables 1 and 2). Most reviews included both drug and alcohol use; two focused on opioid use and six on alcohol (Table 1).

**TABLE 1:** Characteristics of included reviews.

First author, year	Population SUD	Population common mental disorder	Review objectives	Search dates	Number of databases searched	Inclusion/exclusion criteria	Types of study design included	Total number of studies in the review	Number of RCTs relevant to our project	Number of participants in relevant RCTs
Amato, 2011 [27]	SUD drug (opioid)	Depression	To evaluate the effectiveness of any psychosocial plus any agonist maintenance treatment versus standard agonist treatment for opiate dependence.	June 2011	4 plus 2 trial register	Adults > 18 years, opiate addicts undergoing any psychosocial associated with any agonist maintenance intervention. Pregnant women were excluded.	RCTs and controlled clinical trial	35	3	279
Cuijpers, 2023 [28]	SUD any	Depression	To examine the effectiveness of psychological treatments compared with control groups in participants with depression and other comorbid mental health disorders.	January 2022	4	Psychological intervention (primarily targeting either depression, the comorbid disorder, or both) compared with a control condition. Only individual, group and guided self‐help interventions were included. General medical disorders (including dementia) were excluded.	RCTs	35	8	Not reported [3157 in all 35 trials]
Grant, 2021 [29]	SUD (alcohol)	Depression	To evaluate the effectiveness of clinical interventions for improving symptoms of adults with co‐occurring.	December 2020	7	Adult participants (at least 50% were 18 years of age or older) with clinical diagnoses for both SUD (alcohol) and depressive disorder. Clinical interventions intended to improve depressive symptoms or reduce alcohol use.	RCTs (parallel‐group, individually or cluster)	36	9	755
			SUD (alcohol) and depressive disorders.							
Hassan, 2017 [30]	SUD drug (opioid)	Depression, Anxiety	To evaluate studies of pharmacotherapy/psychotherapy for treating symptoms of depression or anxiety in participants receiving opiate agonist treatment.	January 2017	3	Participants on opiate agonist treatment (either methadone or buprenorphine) with anxiety or depression (excluded major depression population but included population with psychotic symptoms or other major psychiatric diagnoses other than SUD).	RCT or non‐RCT	22	4	240
Hides, 2019 [31]	SUD any	Depression	To assess the efficacy of psychological interventions delivered alone or in combination with pharmacotherapy for people diagnosed with comorbid depression and SUD.	February 2019	4 plus Google Scholar and clinical trials registers	RCTs, psychological treatments, diagnosed, comorbid depression and SUD. Studies were included if some of the sample had another disorder (e.g., anxiety); studies with a third disorder were excluded. Those with psychosis, bipolar disorder and intellectual disability were excluded. Comparators were no treatment, delayed treatment, treatment as usual or other psychological treatments.	RCTs	7	6	438
O'Donnell, 2022 [32]	SUD (alcohol)	Depression, major depression	Evaluate the effectiveness of combined digital interventions for comorbid heavy drinking and major depression or clinical depression in community‐dwelling populations.	October 2021	8	Participants with SUD (alcohol) (heavy drinking) and depression ICD‐10 or DSM‐V (major depression disorder, persistent depressive disorder or clinical depression). Intervention: digital interventions, personalised, for SUD (alcohol) and depression.	RCTs	5	5	1503
Riper, 2014 [33]	SUD (alcohol)	Depression, major depression	To evaluate the effectiveness of combining cognitive‐behavioural therapy and motivational interviewing to treat comorbid clinical and subclinical SUD (alcohol) and major depression and estimate the effect of this compared with usual care.	June 2013	3 plus trial register	Effects of cognitive‐behavioural therapy and motivational interviewing on SUD (alcohol), abuse or dependence and depression, compared with treatment as usual or another psychological treatment.	RCT or non‐RCT controlled trials	12	9	1291
Schouten, 2022 [34]	SUD (alcohol)	Depression	To assess the effectiveness of digital interventions addressing depressive symptoms and alcohol use simultaneously amongst people with co‐occurring SUD (alcohol) and depression.	June 2020	7	Participants with SUD (alcohol) and depression, using digital intervention that targeted both depressive symptoms and alcohol use simultaneously.	RCTs	6	6	647
Baker, 2012 [35]	SUD (alcohol)	Mixed common mental disorder (depression, anxiety, panic disorder, phobias)	To evaluate the effectiveness of psychological interventions in people who misuse alcohol with co‐occurring depressive or anxiety disorders.	March 2010	2	People with SUD (alcohol) and depression or anxiety or phobia diagnosis (excluding large proportion psychosis); using treatment manual intervention; report data on alcohol use outcomes.	RCTs	8	8	738
Boniface, 2018 [36]	SUD (alcohol)	Mixed common mental disorder (depression, anxiety, PTSD, social phobia)	To evaluate the effectiveness of brief interventions for alcohol amongst adults with risky alcohol consumption and comorbid mental health conditions.	May 2016	3	SUD (alcohol) and mental health condition (includes serious mental illness). Brief interventions.	RCT	17	11	2421
Cavicchioli, 2018 [37]	SUD any	Mixed common mental disorder (depression, anxiety, eating disorders, PTSD)	To evaluate the effectiveness of mindfulness‐based interventions compared with clinical practise to treat SUD.	August 2017	4	SUD diagnosed by DSM, with or without comorbid disorder. Mindfulness‐based interventions.	RCT or non‐RCT	37	8	412
Chetty, 2023 [38]	SUD any	Mixed (depression, anxiety, PTSD, eating disorders)	To examine the evidence on integrated and non‐integrated treatment outcomes for participants with co‐occurring diagnosis.	Not reported (included studies up to 2018)	10	Adults ≥ 18 years with co‐occurring diagnosis (i.e., SUD and co‐occurring mental health DSM‐IV).	RCTs	11	9	1011
Dugdale, 2019 [39]	SUD any	Mixed common mental disorder (Depression, PTSD)	To evaluate the effectiveness of computer‐based interventions at improving substance misuse and mental health outcomes.	March 2019	6	SUD (disorder or hazardous use) and mental disorder. Age 16+. Computer‐based or Internet‐delivered intervention.	Quantitative, experimental or intervention studies including RCTs, single‐group pre‐ and post‐measures, longitudinal, and cross‐sectional studies	28	19	Not reported [1177 in all 28 RCTs]
Ghetti, 2022 [40]	SUD any	Mixed common mental disorder (depression, anxiety, PTSD)	To compare the effectiveness of music therapy on psychological symptoms, substance craving, motivation for treatment, and motivation to stay clean/sober.	Feb 2021	10	Participants with formal diagnosis of SUD. People with dual diagnoses with mental health problems or learning problems were eligible. Intervention: must be labelled ‘music therapy’, and conducted by a qualified music therapist, compared with Standard care without music therapy.	RCTs, including the first phase of cross‐over trials, and cluster‐RCTs	21	2	24
Glover‐Wright, 2023 [41]	SUD any	Mixed common mental disorder (depression, major depression, anxiety, panic disorder, PTSD)	To evaluate the impact of physically co‐locating specialist services SUD and mental health services.	October 2021	5	Adults with co‐occurring diagnosis of SUD and mental disorder. Outpatient services consisting of either: (i) mental health specialist care co‐located in alcohol and other drug treatment service settings; (ii) alcohol and other drug specialist care co‐located in mental healthcare service settings; or (iii) dedicated co‐occurring diagnosis services in the same service location. comparators—none; either SUD or mental health treatment; non‐co‐located services.	Not specified	28 (10 RCTs, but some serious mental illness)	4	2497
Hesse, 2009 [42]	SUD any	Mixed common mental disorder (depression, anxiety, OCD, PTSD)	To assess integrated treatment of SUD and co‐morbid conditions, such as depression or anxiety.	Not reported (included studies up to 2008)	4	Adults with SUD and depression or anxiety. Comparing integrated non‐somatic treatment for both SUD and depression or anxiety, with treatment focusing on SUD. Interventions with a mixture of somatic and non‐somatic treatments were excluded. Only RCT and published studies, psychiatric symptoms or substance use outcomes.	RCTs	10	10	223
Karapareddy, 2019 [43]	SUD any	Mixed common mental disorder (anxiety, social phobia, PTSD)	To determine whether existing service models are effective in treating combined mental health and SUD and to examine whether an integrated model of service delivery should be recommended.	2015	8 plus trial register	Studies published between 2004 and 2015 in English, outcomes SUD and mental health disorder and/or total costs or treatment costs.	Quantitative/qualitative	12	4	615
Mehta, 2021 [44]	SUD any	Mixed common mental disorder (depression, anxiety, PTSD)	To examine the efficacy of an integrated cognitive‐behavioural intervention delivered to individuals with an alcohol or other drug use disorder and a co‐occurring mental disorder.	December 2019	Several (Cochrane Register and EBSCO)	Adults (age ≥ 18) meeting criteria for alcohol or other drug use disorder and at least one co‐occurring mental health disorder, published in English language between 1990 and 2019, RCTs.	RCTs	15	12	1646
Perry, 2019 [45]	SUD any	Mixed common mental disorder (depression, anxiety, phobias, PTSD)	To assess the effectiveness of interventions for drug‐using offenders with co‐occurring mental health problems in reducing criminal activity or drug use, or both.	February 2019	12	People involved in the criminal justice system with co‐occurring mental health problems and drug misuse problems regardless of gender, age, ethnicity. Included interventions designed to eliminate or prevent relapse to drug use or criminal activity, or both.	RCT	13	7	1197
Henderson, 2020 [46]	SUD any	PTSD	To explore the literature on group interventions, integrated to treat SUD and PTSD, for women in the criminal justice system.	July 2020	5	Adult women with SUD and PTSD who are involved in criminal justice system. Integrated treatment for SUD and PTSD.	RCTs and non‐RCTs	13	6	894
Hien, 2023 [47]	SUD any	PTSD	To evaluate the effectiveness of behavioural and pharmacological therapies for adults with co‐occurring PTSD and alcohol or other drug use disorders.	December 2020	3	Participants with SUD and PTSD, and treated with psychological or pharmacological interventions.	RCTs	36	27	4046
Hien, 2023 [48]	SUD any	PTSD	To evaluate the evidence base of psychotherapy and pharmacological interventions for SUD and PTSD.	August 2019	8	Adults (age 18–75), SUD and PTSD (diagnosed or subthreshold); psychological or pharmacological interventions aimed at SUD or PTSD or both.	Primary studies (RCTs or single‐arm studies)	39	24	Not reported (4046 in all 39 RCTs)
Hill, 2024 [49]	SUD any	PTSD	To examine differences in trauma‐focused and non‐trauma‐focused treatment outcomes for individuals who did and did not endorse baseline cannabis use.	2017	2	Participants with SUD and PTSD. Psychotherapy interventions—trauma focussed. comparator non‐trauma focused. Measured baseline cannabis use.	RCT	4	4	413
Logsdon, 2023 [50]	SUD any	PTSD	To synthesise existing studies on the effectiveness of both trauma‐focused and addiction‐focused eye movement desensitisation and reprocessing for people with SUD.	Not reported, [included studies to 2021]	2 + EBSCO Discovery platform	SUD, eye movement desensitisation and reprocessing, controlled study, outcome of SUD, PTSD, depression or other mental health.	RCT, experimental or quasi‐experimental design with control groups	10	3	66
Molina, 2022 [51]	SUD any	PTSD	To assess the effectiveness of psychological interventions on emotion regulation, PTSD and SUD symptoms in adults with adverse childhood experiences.	April 2019	5	Adults with adverse childhood experiences with SUD and PTSD. Psychological, trauma‐focused or trauma‐informed psychological intervention. Outcomes: treatment effect or implementation. Published after 2008.	RCT or quasi‐experimental	13	8	1230
Roberts, 2022 [52]	SUD any	PTSD	To evaluate psychological treatments for PTSD or PTSD and SUD, when compared to treatment for SUD only or other active treatments.	2021	8	Participants with formal diagnosis for PTSD (ICD‐10) or (DSM‐V), or subthreshold PTSD. Formal diagnosis for SUD according to ICD or DSM (not nicotine). evaluating one or more psychological interventions, in comparison to a control condition or an alternative psychological intervention.	RCTs or cluster RCTs	27	26	2816
Sherman, 2023 [53]	SUD any	PTSD	To examine the effect of seeking safety on comorbid PTSD and SUD.	January 2023	5	Participants with SUD and PTSD seeking safety intervention.	RCT	7	7	363
Simpson, 2021 [54]	SUD any	PTSD	To investigate the efficacy and acceptability of trauma‐focused, non‐trauma‐focused and cognitive‐behavioural manualised SUD therapies for SUD (alcohol) and PTSD.	July 2021	10 and trial registries	Adults ≥ 18 years old with current comorbid SUD and PTSD; cognitive and/or behavioural treatments for both PTSD and SUD.	RCTs	28	25	2805

Abbreviations: PTSD, post‐traumatic stress disorder; RCT, randomised controlled trial; SUD, substance use disorder.

**TABLE 2:** Review outcomes, intervention versus comparator for randomised controlled trials (RCT) eligible for this review.

First author, year	Population SUD	Population common mental disorder	Intervention	Comparator	Method of analysis	Main finding: intervention versus comparator
						Effect size (95% CI) [heterogeneity *I* ^2^] (significant meta‐analyses in bold)
Amato, 2011 [27]	SUD drug (opioid)	Depression	Any psychosocial intervention plus pharmacological (standard clinic counselling, acceptance and commitment therapy, peer support, Supportive‐Expressive Therapy, CBT)	Pharmacological, any agonist treatments alone for opiate maintenance therapy	Meta‐analysis	Depression (SCL −90), SMD 0.02 (−0.28, 0.31) [*I* ^2^ 0%]
Cuijpers, 2023 [28]	SUD any	Depression	Psychological interventions primarily targeting either depression, the comorbid disorder or both (with or without antidepressants)—CBT, psychotherapy	Waiting list, treatment as usual or non‐active control (with or without antidepressants)	Meta‐analysis	**Depression (HAM‐D‐17, BDI, BDI‐II), SMD 0.25 (0.06, 0.43) [*I*** ^**2**^ **25%]**
						**Substance use, SMD 0.25; 95% CI (0.01, 0.50) [*I*** ^**2**^ **58%]**
Grant, 2021 [29]	SUD (alcohol)	Depression	Supportive text messaging plus treatment as usual	Psychological placebo (attention matched) plus treatment as usual	Meta‐analysis	Depression (BDI), SMD −0.17 (−0.74 to 0.39) [*I* ^2^ 0%]
						Alcohol use, SMD −0.14 (−0.70 to 0.43) [*I* ^2^ 0%] [at 1–5 months follow‐up]
Grant, 2021 [29]	SUD (alcohol)	Depression	CBT/brief CBT plus treatment as usual, with or without pharmacological	Treatment as usual (brief supportive psychotherapy)	Meta‐analysis	**Depression (PHQ‐9), SMD −0.84 (−1.05 to −0.63) *p* < 0.001 [*I*** ^**2**^ **0%]**
						**Alcohol use, SMD −0.25 95% CI (−0.47 to −0.04); *p* = 0.021 [*I*** ^**2**^ **64.9%]**
Hassan, 2017 [30]	SUD drug (opiate)	Depression/Anxiety	Behavioural therapy, psychotherapy or CBT plus opiate agonist	Treatment as usual (recovery/relaxation training, group therapy)	Narrative	Depressive symptoms, CBT or psychotherapy better than treatment as usual; behavioural therapy similar results to treatment as usual
Hides, 2019 [31]	SUD any	Depression	Group‐based integrated CBT	Peer support	Meta‐analysis	**Depression (Hamilton Rating Scale for Depression[HDRS]), SMD** −**4.05 95% CI (1.43 to 6.66) significantly favours peer support [*I*** ^**2**^ **0%]** (no significant difference at 6–12 months follow‐up)
						Substance use (PDA), SMD −2.84 95% CI (−8.04 to 2.35) nonsignificant [*I* ^2^ 0%] **(*p* = 0.01 significantly favours Integrated CBT at 6–12 months follow‐up)**
Hides, 2019 [31]	SUD any	Depression	Psychotherapy	Treatment as usual (Brief Supportive Psychotherapy or Psychoeducation)	Meta‐analysis	**Depression (HDRS), SMD** −**0.54 (−1.04 to 0.04) *p* = 0.03 [*I*** ^**2**^ **0%]**
O'Donnell, 2022 [32]	SUD (alcohol)	Depression, major depression	Digital interventions, personalised, for SUD (alcohol) and depression. Includes clinician assisted computer‐based intervention, integrated digital or web‐based interventions	Face‐to‐face manualised brief intervention with or without motivational interview; alcohol only web‐based; attention‐control web‐based	Narrative	For both depression and alcohol use, digital intervention significantly better than comparator at 1 month follow‐up, but non‐significant at 3–6 months.
Riper, 2014 [33]	SUD (alcohol)	Depression, major depression	CBT/motivational interview, some integrated, with or without pharmacological	Treatment as usual/brief treatment (psychosocial counselling and/or medication treatment/peer support)	Meta‐analysis	**Depression (Hospital Anxiety and Depression Scale [HADS], Hamilton Depression Rating Scale [HAM‐D], Beck Depression Inventory [BDI]), Standardised mean difference [SMD] 0.23 (0.07 to 0.39) *p* < 0.01, [*I*** ^**2**^ **11.9%]**
						**Alcohol use (Drinks per drinking day, days abstinent, ≥ 50% reduction, % above harmful threshold, SUD (alcohol)IT), SMD 0.15 (0.03 to 0.28) *p* ≤ 0.05 [*I*** ^**2**^ **38.8%]**
Schouten, 2022 [34]	SUD (alcohol)	Depression	Integrated digital intervention CBT/MI	Any type of control group (e.g., WL, active treatment, assessment only, attention control)	Meta‐analysis	**Depressive symptoms (BDI; Patient Health Questionnaire [PHQ‐9]), SMD at 3 months (0.06, 0.62) *p* = 0.02 [*I*** ^**2**^ **27%]**; at 6 months nonsignificant. Alcohol use (units per day or per week, or alcohol use occasions per day): nonsignificant at 3 months; **at 6 months SMD (0.07, 0.20) *p* = 0.005 (sig favours intervention) [*I*** ^**2**^ **0%]**
Baker, 2012 [35]	SUD (alcohol)	Mixed common mental disorder (depression, anxiety, panic disorder, phobias)	Manual guided (CBT/motivational interview, psychotherapy, manual‐led therapy, integrated cognitive, or sequential behavioural therapy)	Information package or brief supportive therapy	Narrative	CBT/motivational interview significantly improved SUD and depression or anxiety symptoms more than treatment as usual (nonsignificant for psychotherapy)
Boniface, 2018 [36]	SUD (alcohol)	Mixed common mental disorder (depression, anxiety, PTSD, social phobia)	Brief interventions or brief advice at reducing alcohol consumption	Active intervention (e.g., motivational interview/CBT) or minimally active comparator (e.g., assessment only)	Narrative	Brief CBT, motivational interview or support interventions (in many trials, a single session only) were significantly better at reducing alcohol consumption, although not across all RCTs, but had similar effectiveness to digital active interventions
Cavicchioli, 2018 [37]	SUD any	Mixed common mental disorder (depression, anxiety, eating disorders, PTSD)	Mindfulness	Treatment as usual CBT, psychoeducation, peer support, rational thinking skills	Meta‐analysis for PTSD outcome; narrative for other common mental disorder outcomes, and SUD for mixed common mental disorder population	**PTSD significantly favoured intervention SMD 2.38 (−2.67, −2.08) *p* < 0.001 [*I*** ^**2**^ **85.65%]**
						Other common mental disorder no significant treatment group difference (including eating disorders, anxiety, depression)
						SUD outcome, Mindfulness significantly improved craving symptoms, but not abstinence, compared to treatment as usual
Chetty, 2023 [38]	SUD any	Mixed (depression, anxiety, PTSD, eating disorders)	Integrated CBT; prolonged exposure; DBT	Non‐integrated treatment as usual, includes supportive counselling	Narrative	Integrated treatment significantly better than non‐integrated treatment in improving mental health symptoms.
						No significant difference in SUD outcomes
Dugdale, 2019 [39]	SUD any	Mixed common mental disorder (depression, PTSD)	Computer based interventions aimed at mental health (based on CBT, motivational interview, acceptance and commitment therapy, mindfulness or peer support)	Wait‐list, psychoeducation	Narrative	Integrated digital interventions were significantly better than wait‐list in reducing substance use and improving common mental disorder outcomes
						Non‐significant difference from psychoeducation.
Ghetti, 2022 [40]	SUD any	Mixed common mental disorder (depression, anxiety, PTSD)	Music therapy plus treatment as usual—music therapy must be conducted by a qualified music therapist	Treatment as usual (psychotherapy, CBT, support)	Narrative	No significant difference in depressive symptoms or SUD
Glover‐Wright, 2023 [41]	SUD any	Mixed common mental disorder (depression, major depression, anxiety, panic disorder, PTSD)	Co‐location of SUD and mental health services (integrated and co‐located in primary care; co‐located psychiatric care)	Referrals to separate mental health services; community psychiatry and parallel SUD treatment as usual; peer support	Narrative	No significant difference in common mental disorder or SUD outcomes
Hesse, 2009 [42]	SUD any	Mixed common mental disorder (depression, anxiety, OCD, PTSD)	Psychotherapeutic integrated treatment	Treatment as usual peer support, best supportive therapy	Meta‐analysis	At 6 months
						**Depression (SCL −90 or BDI) SMD −0.58, 95% CI (−1.10 to −0.06) *p* = 0.03 [*I*** ^**2**^ **= 46%]**
						**SUD (percent days abstinent) SMD 13.75, 95% CI (0.51, 26.99), *p* = 0.04 [*I*** ^**2**^ **= 17%]**
Hesse, 2009 [42]	SUD any	Mixed common mental disorder (depression, anxiety, OCD, PTSD)	Integrated non‐somatic treatment for SUD and common mental disorder (CBT + SUD treatment program)	Treatment for substance use disorders alone (regular program)	Narrative	No sig diff for social anxiety/PSTD.
						For anxiety/OCD trend towards favouring integrated treatment
						No sig diff, and in some cases alcoholism treatment alone was non‐significant trend superior to alcoholism treatment
Karapareddy, 2019 [43]	SUD any	Mixed common mental disorder (anxiety, social phobia, PTSD)	Integrated service models (acceptance and commitment therapy, compensated work therapy)	Standard care, CWT alone	Narrative	Integrated models of care were more effective than non‐integrated for common mental disorder and SUD
Mehta, 2021 [44]	SUD any	Mixed common mental disorder (depression, anxiety)	ICBT+ treatment as usual	Treatment as usual, for SUD	Meta‐analysis	SUD (days using, proportion days abstinent) SMD 0.240 (−0.163, 0.643), *p* = 0.243 [*I* ^2^ = 87%]
Mehta, 2021 [44]	SUD any	Mixed common mental disorder (PTSD)	ICBT+ treatment as usual	Treatment as usual, for SUD	Meta‐analysis	**SUD (days using, proportion days abstinent) SMD 0.245 (0.002, 0.489) *p* = 0.005 [*I*** ^**2**^ **= 54%]**
Perry, 2019 [45]	SUD any	Mixed common mental disorder (depression, anxiety, phobias, PTSD)	IPT, MI, MBI and CBT, therapeutic community, peer support	Treatment as usual (education, case management) or wait‐list	Narrative	No significant difference for SUD
Henderson, 2020 [46]	SUD any	PTSD	Gender responsive therapy, therapeutic community, seeking support	Treatment as usual, CBT, group therapy	Narrative	Significantly favoured Therapeutic community over CBT for common mental disorder and SUD outcomes; Gender responsive therapy sig favoured for SUD if community‐based, not in prison, not for common mental disorder.
						Support nonsignificant compared to treatment as usual for SUD or common mental disorder
Hien, 2023 [47]	SUD any	PTSD	Trauma focused therapy integrated	Treatment as usual	Outcome model	**PTSD −0.47 (−0.94, −0.01)** (at 12 months, nonsignificant)
						**Alcohol use −0.42 (−0.74, −0.10)** (drug use nonsignificant at 12 months, nonsignificant)
Hien, 2023 [47]	SUD any	PTSD	Trauma focused therapy and pharmacotherapy for SUD	Treatment as usual	Outcome model	**PTSD −0.92 (−1.57, −0.30) (also significant at 12 months)**
						**Alcohol use −1.10 (−1.54, −0.68)** (drug use not significant), **(alcohol use also significant at 12 months)**
Hien, 2023 [48]	SUD any	PTSD	Integrated trauma focused therapy	Integrated non‐trauma‐focused	Meta‐analysis	**PTSD −0.30 (−0.56, −0.04) *p* = 0.022 [*I*** ^**2**^ **= 20.7%]**
						SUD **−**0.17 [−0.45, 0.11] [*I* ^2^ = 31.5%]
Hien, 2023 [48]	SUD any	PTSD	Integrated trauma focused therapy	Treatment as usual psychotherapy	Meta‐analysis	**PTSD −0.43 (−0.68, −0.18) *p* < 0.001 [*I*** ^**2**^ **= 20.7%]**
						SUD **−**0.03 (−0.31, 0.25) [*I* ^2^ = 31.5%]
Hill, 2024 [49]	SUD any	PTSD	Trauma focused therapy, concurrent treatment of PTSD and substance use disorders using prolonged exposure	Treatment as usual, seeking safety, relapse prevention	Narrative	No significant difference for PTSD or SUD
Logsdon, 2023 [50]	SUD any	PTSD	Eye movement desensitisation and reprocessing	Active control	Meta‐analysis	**PTSD symptom 1.426 (0.196, 2.656) *p* = 0.023, [*I*** ^**2**^ **= 80%]**
Molina, 2022 [51]	SUD any	PTSD	Trauma‐focused or seeking safety, or integrated CBT	Treatment as usual, or WL	Narrative	PTSD One RCT sig favoured trauma focused intervention, others trend
						SUD trend favouring trauma‐focused treatment for 3 RCTs, other RCTs nonsignificant
Roberts, 2022 [52]	SUD any	PTSD	Integrated CBT + treatment as usual for SUD	Treatment as usual for SUD only	Meta‐analysis	PTSD −0.24 (−0.51, 0.03) [*I* ^2^ = 33%]
						Alcohol use 0.02 (−0.19, 0.24); Substance use −0.08 (−0.30, −0.13) [*I* ^2^ = 0%]
Roberts, 2022 [52]	SUD any	PTSD	Present‐focused treatments + treatment as usual for SUD (seeking safety or coping skills)	Treatment as usual for SUD only	Meta‐analysis	PTSD −0.02 (−0.16, 0.12). [*I* ^2^ = 0%]
						Alcohol use −0.21 (−0.67, 0.25); Substance use 0.13 (−0.42, 0.16) [*I* ^2^ = 67%]
Sherman, 2023 [53]	SUD any	PTSD	Seeking safety	Treatment as usual, community care, individual therapy or education	Meta‐analysis	PTSD at 6 months −0.17 (−0.36, 0.02) (*p* = 0.86) [*I* ^2^ = 0%]
						SUD at 3 months −0.20 (−0.88, 0.48) (*p* = 0.13) [*I* ^2^ = 51%]
Simpson, 2021 [54]	SUD any	PTSD	Prolonged exposure, trauma‐focused CBT	Treatment as usual, wait‐list	Meta‐analysis	**PTSD 0.29 (0.07, 0.52) [*I*** ^**2**^ **= 33%]** [later follow‐up nonsignificant]
						SUD −0.11 [−0.34, 0.11] [*I* ^2^ = 25%]
Simpson, 2021 [54]	SUD any	PTSD	CBT, or integrated CBT, seeking safety, web based peer support	Treatment as usual, wait‐list		PTSD 0.11 [−0.03, 0.26] [*I* ^2^ = 5%]
						SUD 0.12 [−0.03, 0.28] [*I* ^2^ = 27%]

Abbreviations: CBT, cognitive behavioural therapy; CI, confidence interval; DBT, dialectical behaviour therapy; OCD, obsessive‐compulsive disorder; PTSD, post‐traumatic stress disorder; SUD, substance use disorder.

The methodological quality of the reviews was generally good (Table 3). All reviews had clear review questions, reported inclusion criteria, and had appropriate search sources. The main area of concern was the lack of assessment for publication bias, and unclear reporting of methods concerning whether critical appraisal was undertaken independently by two or more reviewers, or whether methods to minimise errors in data extraction were used. Due to heterogeneity in population, interventions, comparators and outcomes it was not possible to quantify any trends between the review quality and significance of outcomes reported.

**TABLE 3:** Critical appraisal of included systematic reviews.

First author, year	1. Is the review question clearly and explicitly stated?	2. Were the inclusion criteria appropriate for the review question?	3. Was the search strategy appropriate?	4. Were the sources and resources used to search for studies adequate?	5. Were the criteria for appraising studies appropriate?	6. Was critical appraisal conducted by two or more reviewers independently?	7. Were there methods to minimise errors in data extraction?	8. Were the methods used to combine studies appropriate?	9. Was the likelihood of publication bias assessed?	10. Were recommendations for policy and/or practise supported by the reported data?	11. Were the specific directives for new research appropriate?
Amato, 2011 [27]	Yes	Yes	Yes	Yes	Yes	Yes	Yes	Yes	Yes	Yes	Yes
Baker, 2012 [35]	Yes	Yes	Yes	Yes	Yes	Yes	Yes	NA	No	Yes	Yes
Boniface, 2018 [36]	Yes	Yes	Yes	Yes	Yes	Yes	Yes	Yes	Yes	Yes	Yes
Cavicchioli, 2018 [37]	Yes	Yes	Yes	Yes	Yes	Unclear	Unclear	Yes	Yes	Yes	Yes
Chetty, 2023 [38]	Yes	Yes	Yes	Yes	Yes	Yes	Unclear	Yes	No	Yes	Yes
Cuijpers, 2023 [28]	Yes	Yes	Yes	Yes	Yes	Yes	Unclear	Yes	Yes	Yes	Yes
Dugdale, 2019 [39]	Yes	Yes	Yes	Yes	Yes	Unclear	Yes	Yes	No	Yes	Yes
Ghetti, 2022 [40]	Yes	Yes	Yes	Yes	Yes	Yes	Yes	Yes	Yes	Yes	Yes
Glover‐Wright, 2023 [41]	Yes	Yes	Yes	Yes	Yes	Unclear	Unclear	Yes	No	Yes	Yes
Grant, 2021 [29]	Yes	Yes	Yes	Yes	Yes	Yes	Yes	Yes	Yes	Yes	Yes
Hassan, 2017 [30]	Yes	Yes	Unclear	Yes	Yes	Yes	Yes	NA	Unclear	Yes	Yes
Henderson, 2020 [46]	Yes	Yes	Yes	Yes	Yes	Unclear	Unclear	Yes	No	Yes	Yes
Hesse, 2009 [42]	Yes	Yes	Yes	Yes	NA	NA	No	Yes	No	Yes	Yes
Hides, 2019 [31]	Yes	Yes	Yes	Yes	Yes	Yes	Yes	Yes	Yes	Yes	Yes
Hien, 2023 [47]	Yes	Yes	Yes	Yes	Yes	Yes	Unclear	Yes	Yes	Yes	Yes
Hien, 2023 [48]	Yes	Yes	Yes	Yes	Yes	Yes	Yes	Yes	Unclear	Yes	Yes
Hill, 2024 [49]	Yes	Yes	Yes	Yes	NA	NA	Unclear	Yes	No	Yes	Yes
Karapareddy, 2019 [43]	Yes	Yes	Yes	Yes	NA	NA	Unclear	Yes	Yes	Yes	Yes
Logsdon, 2023 [50]	Yes	Yes	Yes	Yes	Yes	Yes	Yes	Yes	Yes	Yes	Yes
Mehta, 2021 [44]	Yes	Yes	Yes	Yes	Yes	Unclear	Yes	Yes	Yes	Yes	Yes
Molina, 2022 [51]	Yes	Yes	Yes	Yes	Yes	Yes	Yes	NA	No	Yes	Yes
O'Donnell, 2022 [32]	Yes	Yes	Yes	Yes	Yes	Yes	Yes	Yes	No	Yes	Yes
Perry, 2019 [45]	Yes	Yes	Yes	Yes	Yes	Yes	Yes	Yes	Yes	Yes	Yes
Riper, 2014 [33]	Yes	Yes	Unclear	Yes	Yes	Yes	Unclear	Yes	Yes	Yes	Yes
Roberts, 2022 [52]	Yes	Yes	Yes	Yes	Yes	Yes	Yes	Yes	No	Yes	Yes
Schouten, 2022 [34]	Yes	Yes	Yes	Yes	Yes	Yes	Yes	Yes	No	Yes	Yes
Sherman, 2023 [53]	Yes	Yes	Yes	Yes	Yes	Yes	Yes	Yes	No	Yes	Yes
Simpson, 2021 [11]	Yes	Yes	Yes	Yes	Yes	Yes	Yes	Yes	Yes	Yes	Yes

Abbreviation: NA, not applicable.

Review evidence was found for the following categories of interventions: cognitive behavioural therapy; peer support; motivational interview; supportive counselling; psychotherapy; behavioural therapy including dialectical behavioural therapy; seeking safety; trauma‐focused therapy; acceptance and commitment therapy; self‐management support; contingency management, that is incentivised or compensated work therapy; mindfulness; eye movement desensitisation and reprocessing; and music therapy. Some of the interventions were delivered via computer or text message (digital interventions), and some were delivered as brief interventions (from as little as a single session). There was also a review investigating the co‐location of services.

There was some overlap of RCTs between Hesse et al. [42], Hides et al. [31] and Mehta et al. [44]; between O'Donnell et al. [32] and Schouten et al. [34]; and Hassan et al. [30] and Amato et al. [27]; Hien et al. [47] and Hien et al. [48]; Molina et al. [51] and Mehta et al. [44]; Simpson et al. [54], Sherman et al. [53] and Roberts et al. [52]. However, all these reviews also included RCTs that did not overlap with other reviews.

The methodological quality of the included RCTs within the reviews varied. Some RCTs did not report key information. The studies with an overall high risk of bias were mainly due to a high dropout rate, failure to meet power calculation estimates, selection bias, use of only self‐report outcomes, lack of blinding, or had meaningful differences in baseline characteristics. However, it should be noted that the inability to blind participants and assessors, and the use of self‐report measures is common in psychological studies. Although it was not possible to assess if the methodological quality of the studies impacted the direction of the results, Cuijpers et al. [28] and Boniface et al. [36] noted that limiting the studies to those with a low risk of bias resulted in a non‐significant effect size. Whilst Mehta et al. [44] found studies with a low risk of bias had greater effects on SUD outcomes, study quality was not related to effect size variability for PTSD outcomes. In Hein et al. [47] the effect size on PTSD symptom severity at 12 months was greater when restricted to studies with low or moderate risk of bias but showed no difference in effect size greater than 0.06 on other outcomes. Amato et al. [27] performed a sensitivity analysis including and excluding studies at high risk of bias, for retention in treatment and substance use, but this did not lead to a change in results. Many reviews were unable to perform sensitivity analysis to explore the impact of bias on effect size due to the small number of included studies and clinical heterogeneity limiting the possibility to pool data. Overall, due to variabilities in the included studies and outcomes measured in the reviews, it was difficult to highlight any trends between the methodological quality of the studies and the type of mental health disorder, type of substance use disorder or by intervention type.

### 3.2 Effectiveness

There was much heterogeneity both between reviews, and between the RCTs within the reviews. There was heterogeneity in populations (type of common mental disorder and severity), interventions and outcome measures, as well as in settings (mostly outpatient services, some inpatient, some services for prisoners or veterans), how interventions or comparators were delivered, and treatment intensity and retention. Most reviews stated the results were not generalisable across all populations or settings. Additionally, within the treatment group, most of the interventions (covering all intervention types) and many of the active comparators studied resulted in some improvement for patients on substance use outcomes and/or mental health outcomes. Many reviews pointed out that RCTs had small sample sizes. Treatment as usual treated both common mental disorder and SUD, but differed between studies, and was not always clearly described. This made it impossible to reach an overall conclusion about which therapies were best overall.

Comparisons between treatment groups found by the included reviews are summarised in Table 2. The following interventions were found to have an advantage over control, for both common mental disorder and SUD outcomes: cognitive behavioural therapy [29, 33, 34, 35, 39]; peer support [39]; motivational interview; depression [33, 34, 35, 39]; psychotherapy [28, 31, 42]; trauma focused therapy [47]; acceptance and commitment therapy [39]; contingency management [43]; and mindfulness [37, 39]. There was also evidence of a significant advantage over treatment as usual for both common mental disorder and SUD outcomes for brief interventions [36], or interventions delivered digitally [32, 34, 39]. There was a variety of interventions within each category, and not all RCTs within these categories found a significant difference from treatment as usual. Some reviews found that therapies improved common mental disorder but not SUD [30, 31, 54]. A review of eye movement desensitisation and reprocessing in PTSD did not measure SUD, but found significantly greater improvement in PTSD compared with treatment as usual [50]. Only one review was found for arts therapy, and this found music therapy had similar effectiveness to treatment as usual for SUD or depression outcomes [40].

Overall, evidence indicated (Table 2) that the following interventions were more beneficial than treatment as usual for both SUD and common mental disorder. Depression and SUD outcomes were improved by cognitive‐behavioural therapy, motivational interviewing, psychotherapy, support, and interventions delivered digitally. Anxiety and SUD outcomes were improved by cognitive‐behavioural therapy, motivational interviewing, mindfulness, digital interventions. PTSD and SUD outcomes were improved by cognitive‐behavioural therapy, motivational interviewing, peer support, behavioural therapy, seeking safety, trauma‐focussed therapy, eye movement desensitisation and reprocessing. Mixed mental health and SUD outcomes were improved by cognitive‐behavioural therapy, motivational interviewing, peer support, contingency management, mindfulness, support, acceptance and commitment therapy, and also brief interventions.

Integrated treatment involves the treatment of co‐occurring diagnoses simultaneously, with co‐ordination of services for comorbid disorders, This may involve a range of service providers and health professionals. For comparison of integrated treatment with parallel treatment (of the two separate disorders), there was evidence favouring treatment of both substance use and mental health disorders for integrated acceptance and commitment therapy plus contingency management [43], integrated trauma‐focussed therapy [47], integrated psychotherapy [42]. Evidence favouring integrated treatment for either substance use, or mental health disorders was found for behavioural or trauma‐focussed therapy [35, 38, 48]. Integrated behavioural therapy was not found to differ significantly from peer support for SUD outcomes, nor for social anxiety or PTSD outcomes but had a non‐significant trend towards improving obsessive compulsive disorder or anxiety symptoms [42, 44]. Integrated cognitive behavioural therapy was equally effective as sequential treatment for panic disorder/agoraphobia [35].

Integrated digital interventions (based on cognitive behavioural therapy, motivational interviewing, acceptance and commitment therapy, cognitive restructuring, mindfulness, or peer support) were significantly better than wait‐list (but not inpatient treatment) in reducing substance use and improving common mental disorder outcomes, more so when computer‐based intervention was combined with therapist support [39]. Integrated co‐located services and parallel services had similar effectiveness for SUD and mixed mental disorder diagnoses [41]. Both integrated and parallel treatments led to reductions in substance use and related harms, mental health symptoms and decreased emergency department presentations or hospital admissions [41]. There were differences between services beyond being integrated or non‐integrated, and so there may have been other factors influencing outcomes (e.g., setting in primary care versus community, contingency management, variety of professionals providing the service).

### 3.3 Treatment Retention

Few of the included reviews reported comparative treatment retention measures. Where treatment retention or attrition was compared between intervention and treatment as usual, there were similar rates between groups [27, 31, 37, 40, 54]. Attrition rates were 50% or more in some cases [32, 51]. Digital interventions may have higher attrition than face‐to‐face [35, 39], it may be that participants engaged in digital interventions that would not have engaged with conventional treatment [32]. The reason for attrition was not explicit, and may represent a lack of therapeutic alliance, relapse or no longer requiring treatment [35, 39]. Co‐location of SUD and mental health services was associated with improved treatment engagement and shorter time between referral and start of treatment [41]. Integrated treatment was associated with better treatment attendance in some cases, but not all trials reported [31, 38]. There was a non‐significant trend favouring integrated treatment over non‐integrated or sequential [31, 52] or SUD treatment alone [42].

## 4 Discussion

The umbrella review identified 28 reviews, of good methodological quality, investigating psychosocial treatments for SUD and common mental disorders. Of the categories of interventions searched for, no systematic review evidence was identified for the interventions of supportive housing, case management, yoga or exercise. Most evidence was for depression, anxiety or post‐traumatic stress disorder, with little evidence for other types of mental disorders such as eating disorders or phobias. Whilst the methodological quality of the reviews was good, the methodological quality of the included RCTs within the reviews varied. Where there was an overall high risk of bias, this was mainly due to a high dropout rate, being under‐powered, selection bias, use of only self‐report outcomes, lack of blinding, or meaningful differences in baseline characteristics.

All categories of psychosocial intervention for which evidence was identified, and many of the treatment as usual comparators studied, resulted in improvement for patients on substance use outcomes and/or mental health outcomes. There was often significant improvement in patient outcomes for both trial arms, even where there was no significant treatment effect. Integrated treatment (treating both SUD and mental disorder) was usually better than treating one condition alone, and sometimes better than parallel treatments (separate, uncoordinated services for SUD, and mental disorder). Integrated treatment was not any more harmful than treating a single disorder. There was limited evidence assessing sequential treatment (with either SUD or mental disorder treated first) but this suggested it was similarly effective to integrated treatment.

### 4.1 Strengths and Limitations of the Review

The eligibility was limited to systematic reviews of RCTs in English language, so studies of interventions for comorbid populations may have been missed. Good quality systematic reviews of RCT evidence were identified supporting psychosocial interventions for the treatment of substance use disorders and common mental health disorders.

### 4.2 Limitations of Included Literature

There was much heterogeneity both between reviews, and between the RCTs within the reviews. There was heterogeneity in interventions and comparators (delivery mode, intensity, duration, adherence); populations diagnoses (diagnostic criteria, types of substance for SUD, baseline severity); and demographics (age, sex, veteran, homelessness); settings (inpatient, outpatient); and outcome measures used. Most reviews discussed that studies aren't generalisable across all SUD and mental disorder comorbid populations, and treatment settings. Treatment as usual varied between studies, in type, intensity and duration, and was not always clearly described. Difficulties of RCTs included small sample sizes, high dropout rates, and the nature of the intervention and comparator groups led to an impossibility of blinding. High dropout rates led to failure to meet power calculation estimates, and difficulty with long‐term follow‐up; in practise follow‐up was not often beyond immediately post‐treatment.

### 4.3 Implications for Policy, Practise and Future Research

Due to data limitations, it was not possible to identify the most effective type of intervention; therefore there are no recommendations for policy or practise. It appears that integration (by co‐ordination or co‐location of different services and types of health professional) is of benefit.

Future research is needed comparing integrated with parallel or sequential treatment, including study of the order in which sequential treatment is delivered. It would be beneficial to have research with follow‐up of 6 months or longer, and sample size large enough to encompass dropout. Whilst blinding of participants and clinicians is usually impossible for this type of study, outcome assessors could be blinded. Outcomes should include both blinded assessment of substance use and mental health outcomes, and service user views on acceptability and on barriers to access and engagement.

## 5 Conclusions

The review indicated that integrated treatment was usually better than treating one condition alone, and sometimes better than parallel treatments. Any category of psychosocial intervention for which evidence was found resulted in some improvement for service users. More evidence is needed, particularly assessing sequential treatment.

## Author Contributions

**Emma L. Simpson:** conceptualisation, methodology, data curation, formal analysis, project administration, validation, writing – original draft preparation. **Munira Essat:** conceptualisation, writing – review and editing, data curation, formal analysis, validation. **Ruth Wong:** conceptualisation, methodology, writing – review and editing. **Sarah Stacey:** conceptualisation, formal analysis, writing – review and editing. **Edward Day:** conceptualisation, formal analysis, writing – review and editing. **Emma L. Simpson, Munira Essat, Ruth Wong, Sarah Stacey, Edward Day:** agreement to be accountable for all aspects of the work in ensuring that questions related to the accuracy or integrity of any part of the work are appropriately investigated and resolved.

## Conflicts of Interest

The authors declare no conflicts of interest.

## Data Availability

This review was part of a larger project on clinical and cost‐effectiveness, which will be published as a report in the National Institute for Health and Care Research journals series. A map showing the availability of reviews by intervention was created in EPPI‐reviewer [55, 56] with the support of Zak Ghouze from EPPI‐reviewer https://tinyurl.com/35pmhve2.

## Appendix A

Medline search strategy.

**Ovid MEDLINE(R) ALL 1946 to February 16, 2024**.

19 February 2024

2141 records

**Table:**

#	Searches	Results
1	((comorbid* or “co morbid*” or coexist* or “co exist*” or concur* or cooccur* or “co occur*”) adj2 mental* adj2 (condition* or disease* or disorder* or disturbanc* or ill*)).tw.	2281
2	dual diagnosis.mp.	2000
3	1 or 2	4184
4	Comorbidity/and Mental Health/	1124
5	Mood Disorders/or Depression/	170,414
6	((depressi* or affective) adj symptom*).tw.	85,177
7	((depressi* or mood or affective or cognitiv* or adjustment) adj3 disorder*).tw.	120,482
8	dysthymi*.tw.	3333
9	exp Anxiety Disorders/	92,451
10	(anxi* or gad).tw.	299,498
11	exp Obsessive‐Compulsive Disorder/	17,017
12	(obsess* or compulsi* or ocd).tw.	34,621
13	(panic* or agoraphobi*).tw.	25,059
14	Stress Disorders, Post‐Traumatic/	42,545
15	((posttrauma* or post‐trauma* or post trauma*) adj3 (stress* or disorder* or psych* or symptom*)).tw.	47,664
16	(ptsd or stress disorder* or combat disorder*).tw.	46,700
17	Borderline Personality Disorder/	8217
18	((borderline or border‐line or “border line” or unstab* or instab*) adj3 (state* or personalit*)).tw.	11,387
19	(eupd or bpd).tw.	12,885
20	((emotion* or affect*) adj3 (dysregulat* or function* or label* or modulat* or reactive* or regulat*)).tw.	115,102
21	Body Dysmorphic Disorders/	1291
22	(body adj3 dysmorphi*).tw.	1565
23	exp Phobic Disorders/	14,347
24	phobi*.tw.	12,921
25	Antisocial Personality Disorder/	10,544
26	((anti‐social or “anti social” or antisocial) adj3 (disorder* or syndrome*)).tw.	2715
27	aspd.tw.	606
28	or/4–27	756,618
29	((substance* or drug* or narcotic* or alcohol*) adj2 (use* or using or addict* or consum* or depend* or disorder* or habit* or misuse* or misusing or withdraw*)).tw.	377,368
30	(amphetamine* or benzodiazepine* or alcohol* or cannabis or cocain* or crack or heroin or ketamine or marijuana or mdma or methadone or methamphetamine* or opiate* or opioid* or opium or phencyclidine).tw,kf.	712,251
31	(stimulant* or psychostimulant* or sedative* or “psychoactive substance*” or nps).tw.	120,705
32	29 or 30 or 31	997,646
33	28 and 32	73,875
34	3 or 33	77,235
35	(MEDLINE or systematic review).tw. or meta analysis.pt.	458,381
36	34 and 35	2646
37	exp Adult/	8,021,015
38	(teen* or youth* or adolescen* or juvenile* or youngster* or first‐grader* or second‐grader* or third‐grader* or fourth‐grader* or fifth‐grader* or sixth‐grader* or seventh‐grader* or highschool* or ((secondary or high*) adj2 (school* or education))).tw. or Adolescent/	2,473,519
39	(child* or stepchild* or step‐child* or kid or kids or girl or girls or boy or boys or teen* or youth* or youngster* or adolescent* or adolescence or preschool* or pre‐school* or kindergarten* or school* or juvenile* or minors or p?ediatric*).tw. or exp. Child/or exp. Infant/	3,823,333
40	38 or 39	4,803,683
41	40 not (40 and 37)	2,834,723
42	36 not 41	2228
43	limit 42 to english language	2141
